# Supplementary material for: Bacillus subtilis and Bifidobacteria bifidum Fermentation Effects on Various Active Ingredient Contents in Cornus officinalis Fruit
Source: Molecules. 2023 Jan 19;28(3):1032. doi: 10.3390/molecules28031032 (PMC9920020; doi:10.3390/molecules28031032)
Supplement: Supplementary file 1 [file molecules-28-01032-s001.zip › molecules-2096559-supplementary.pdf]

molecules

MDPI

Supplementary Materials

Bacillus subtilis and *Bifidobacteria bifidum* Fermentation Effects on Various Active Ingredient Contents in *Cornus officinalis* Fruit

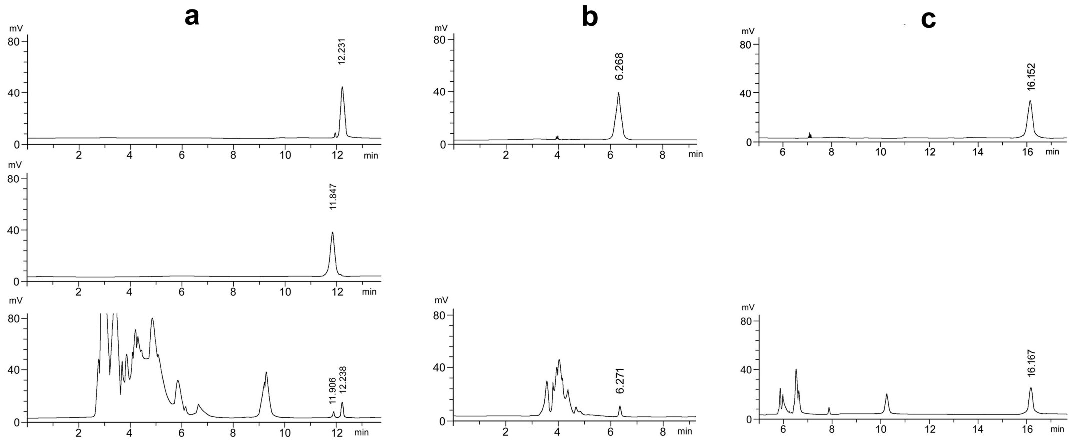

Figure S1. High-performance liquid chromatography for the active compounds in *C. officinalis* fruit culture broth and the corresponding standards. (**a**) Ursolic acid and oleanolic acid. The sample was fermented by *B. subtilis*. (**b**) Gallic acid. The sample was fermented by *B. bifidum*. (**c**) Loganin. The sample was fermented by *B. bifidum*.

Table S1. Ursolic acid contents in *Cornus officinalis* fruit (COF) culture broth at different concentrations and fermentation times.

COF Density (g/L)	Fermentation Time (h)							
	0	6	12	18	24	30	36	42
6 (Bs)	12.32 ± 0.65 a	11.73 ± 0.81 a	10.16 ± 1.90 a	7.71 ± 1.49 b	7.20 ± 1.21 b	7.22 ± 2.31 b	7.11 ± 1.75 b	7.23 ± 0.94 b
12 (Bs)	22.69 ± 0.87 a	21.84 ± 2.08 a	18.64 ± 1.77 b	14.67 ± 1.09 c	13.38 ± 1.41 c	13.90 ± 0.19 c	13.47 ± 0.55 c	13.38 ± 1.43 c
24 (Bs)	43.77 ± 4.40 a	41.61 ± 2.18 a	37.94 ± 3.67 ab	32.37 ± 3.01 bc	29.96 ± 3.33 c	29.16 ± 2.58 c	30.22 ± 4.92 c	29.63 ± 2.94 c
6 (Bb)	13.12 ± 3.56 a	12.63 ± 2.53 a	12.56 ± 1.55 a	12.83 ± 4.65 a	13.03 ± 0.71 a	13.28 ± 2.91 a	12.85 ± 1.66 a	13.10 ± 2.98 a
12 (Bb)	26.40 ± 4.79 a	27.53 ± 3.83 a	26.89 ± 2.88 a	26.76 ± 2.12 a	28.17 ± 5.00 a	27.07 ± 4.68 a	27.23 ± 3.09 a	26.99 ± 3.71 a
24 (Bb)	59.33 ± 6.84 a	58.90 ± 6.81 a	58.80 ± 8.65 a	58.35 ± 11.15 a	60.24 ± 6.11 a	58.42 ± 8.03 a	60.52 ± 6.33 a	58.35 ± 3.40 a

The different letters that follow each value indicate significant differences ($p < 0.05$) in mean values. We compared the different mean values in the rows, and we detected the significant differences ($p < 0.05$) of the mean values using the T-test. We express the ursolic acid content in milligrams per 1000 mL of culture broth. Bs: *Bacillus subtilis*; Bb: *Bifidobacterium bifidum*. The difference in the initial ursolic acid contents between the *B. subtilis* and *B. bifidum* fermentation was due to the usage of two different batches of COF.

Table S2. Oleanolic acid contents in *Cornus officinalis* fruit (COF) culture broth at different concentrations and fermentation times.

COF Density (g/L)	Fermentation Time (h)							
	0	6	12	18	24	30	36	42
6 (Bs)	2.46 ± 0.41 a	2.29 ± 0.06 ab	2.10 ± 0.03 b	1.87 ± 0.03 bc	1.45 ± 0.06 d	1.44 ± 0.1 d	1.41 ± 0.11 d	1.47 ± 0.07 d
12 (Bs)	4.80 ± 0.16 a	4.55 ± 0.17 a	4.10 ± 0.04 b	3.78 ± 0.16 bc	3.55 ± 0.20 c	3.54 ± 0.22 c	3.52 ± 0.18 c	3.55 ± 0.29 c
24 (Bs)	9.87 ± 0.30 a	9.72 ± 0.34 a	9.52 ± 0.30 a	8.81 ± 0.21 b	7.93 ± 0.29 c	7.99 ± 0.33 c	7.94 ± 0.29 c	7.94 ± 0.31 c
6 (Bb)	2.72 ± 0.30 a	2.70 ± 0.31 a	2.80 ± 0.17 a	2.69 ± 0.37 a	2.76 ± 0.12 a	2.74 ± 0.15 a	2.70 ± 0.35 a	2.80 ± 0.10 a
12 (Bb)	5.71 ± 0.43 a	5.64 ± 0.48 a	5.80 ± 0.53 a	5.76 ± 0.34 a	5.71 ± 0.26 a	5.55 ± 0.42 a	5.60 ± 0.20 a	5.68 ± 0.27 a
24 (Bb)	10.50 ± 1.12 a	10.79 ± 0.62 a	10.82 ± 0.71 a	10.44 ± 0.48 a	10.69 ± 0.57 a	10.55 ± 0.45 a	10.84 ± 0.30 a	10.70 ± 0.23 a

The different letters that follow each value indicate significant differences ($p < 0.05$) in mean values. We compared the different mean values in the rows, and we detected the significant differences ($p < 0.05$) of the mean values using the T-test. We express the oleanolic acid content in milligrams per 1000 mL of culture broth. Bs: *Bacillus subtilis*; Bb: *Bifidobacterium bifidum*. The difference in the initial oleanolic acid contents between the *B. subtilis* and *B. bifidum* fermentation was due to the usage of two different batches of COF.

Table S3. Loganin content in *Cornus officinalis* fruit (COF) culture broth at different concentrations and fermentation times.

COF Density (g/L)	Fermentation Time (h)							
	0	6	12	18	24	30	36	42
6(Bs)	51.10 ± 2.05a	51.37 ± 2.89a	51.92 ± 1.79a	51.37 ± 3.09a	51.57 ± 3.31a	52.30 ± 3.83a	52.00 ± 4.68a	52.66 ± 0.655a
12(Bs)	106.22 ± 4.32a	104.26 ± 3.60a	104.17 ± 1.53a	106.27 ± 2.06a	106.07 ± 4.13a	104.53 ± 3.27a	106.46 ± 3.91a	104.93 ± 5.53a
24(Bs)	217.57 ± 1.62a	218.66 ± 8.59a	216.82 ± 7.10a	216.45 ± 3.37a	216.43 ± 8.30a	219.71 ± 8.68a	219.42 ± 2.26a	219.56 ± 7.25a
6(Bb)	47.30 ± 3.93a	47.58 ± 4.47a	47.90 ± 2.41a	48.18 ± 4.93a	47.93 ± 2.36a	47.80 ± 1.11a	47.78 ± 3.17a	48.37 ± 3.64a
12(Bb)	96.56 ± 7.50a	97.21 ± 3.50a	95.82 ± 5.52a	97.15 ± 1.19a	97.44 ± 2.58a	95.73 ± 6.07a	97.42 ± 2.76a	97.17 ± 2.14a
24(Bb)	200.98 ± 8.30a	200.42 ± 6.48a	201.13 ± 7.60a	197.19 ± 3.26a	196.96 ± 10.50a	202.90 ± 8.58a	198.56 ± 7.27a	196.08 ± 3.87a

The different letters that follow each value indicate significant differences ($p < 0.05$) in mean values. We compared the different mean values in the rows, and we detected the significant differences ($p < 0.05$) of the mean values using the T-test. We express the loganin content in milligrams per 1000 mL of culture broth. Bs: *Bacillus subtilis*; Bb: *Bifidobacterium bifidum*. The difference in the initial loganin contents between the *B. subtilis* and *B. bifidum* fermentation was due to the usage of two different batches of COF.

Table S4. Gallic acid contents in *Cornus officinalis* fruit (COF) culture broth at different concentrations and fermentation times.

COF Density (g/L)	Fermentation Time (h)							
	0	6	12	18	24	30	36	42
6 (Bs)	16.96 ± 3.43 c	23.73 ± 3.20 bc	28.35 ± 3.87 ab	33.36 ± 3.17 a	34.96 ± 4.95 a	34.83 ± 34.56 a	34.56 ± 2.64 a	34.19 ± 4.60 a
12 (Bs)	35.96 ± 3.83 c	45.73 ± 3.40 b	47.68 ± 1.69 b	52.69 ± 4.31 ab	57.30 ± 5.08 a	57.17 ± 6.10 a	59.23 ± 5.36 a	58.19 ± 3.98 a
24 (Bs)	71.13 ± 3.36 c	74.46 ± 2.15 c	82.48 ± 4.24 b	95.84 ± 2.21 a	96.68 ± 4.56 a	97.49 ± 5.63 a	96.68 ± 2.01 a	97.14 ± 4.50 a
6 (Bb)	16.00 ± 2.64 c	19.18 ± 2.98 c	24.83 ± 3.41 b	28.04 ± 3.05 ab	29.78 ± 3.68 a	29.71 ± 2.79 a	28.33 ± 2.20 a	29.01 ± 1.40 a
12 (Bb)	32.60 ± 3.90 c	36.01 ± 3.99 bc	42.38 ± 1.44 b	50.47 ± 3.55 a	54.44 ± 5.58 a	55.11 ± 5.76 a	54.78 ± 4.73 a	54.56 ± 3.17 a
24 (Bb)	69.50 ± 2.66 d	72.67 ± 6.48 d	84.27 ± 7.02 c	96.32 ± 4.70 b	104.59 ± 6.14 ab	107.10 ± 6.86 a	107.29 ± 5.49 a	106.55 ± 6.70 a

The different letters that follow each value indicate significant differences ($p < 0.05$) in mean values. We compared the different mean values in the rows, and we detected the significant differences ($p < 0.05$) in the mean values using the T-test. We express the gallic acid content in milligrams per 1000 mL of culture broth. Bs: *Bacillus subtilis*; Bb: *Bifidobacterium bifidum*. The difference in the initial gallic acid contents between the *B. subtilis* and *B. bifidum* fermentation was due to the usage of two different batches of COF.
